# Quantifying of highly radioactive and radiotoxic polonium-210 intake from cannabis (*Cannabis sativa L.*): impacts of different smoking and vaporization techniques

**DOI:** 10.1007/s11356-024-35263-w

**Published:** 2024-10-15

**Authors:** Jarosław Wieczorek, Alicja Boryło

**Affiliations:** Department of Chemistry and Radiochemistry of Environment, Faculty of Chemistry, the University of Gdansk, Gdansk, Poland

**Keywords:** Polonium, Cannabis, Dose, Smoke, Desorption

## Abstract

This study investigates the radiation dose that humans receive from naturally occurring uranium-series radionuclides, with a focus on alpha-emitting polonium-210 (^210^Po), which has a half-life of 138.4 days. Polonium is efficiently absorbed by living organisms and is estimated to contribute approximately 7% of the total effective dose from ingested natural internal radiation. The primary source of atmospheric ^210^Po is the decay of radon-222 (^222^Rn), which precipitates onto land and water surfaces, including crops. In *Cannabis sativa*, ^210^Po accumulates primarily through direct deposition on leaf surfaces and from fertilizers, particularly phosphate fertilizers. This study examined 10 samples of dried hemp (*Cannabis sativa* L.) legally available in Poland, assessing ^210^Po concentrations through radiochemical analysis. Various consumption methods were analyzed, including smoking with a glass pipe, a water pipe (bong), a cigarette (joint or spliff), and vaporization. The desorption of ^210^Po during combustion and vaporization was measured, revealing significant differences based on the method used. Results showed that smoking with a glass pipe resulted in the highest desorption of ^210^Po (approximately 80%), while water pipe and blunt smoking exhibited lower desorption rates (around 40%). The efficiency of ^210^Po adsorption in filters varied, with water filters showing about 8% absorption and cellulose filters about 20%. Vaporization results indicated that the desorption of ^210^Po increased with temperature. These findings underscore the importance of considering the method of consumption when calculating the radiation dose from ^210^Po, as the differences can significantly impact the accuracy of dose assessments. The study highlights the need for further research to optimize safety guidelines for cannabis consumption in relation to radioactive contaminants.

**Supplementary Information:**

The online version contains supplementary material available at 10.1007/s11356-024-35263-w.

## Introduction

The radiation dose that humans receive includes contributions from naturally occurring uranium-series radionuclides that accumulate in the body, particularly the alpha-emitting ^210^Po (with a physical half-life of 138.4 days) (UNSCEAR 1993). Polonium, in particular, is efficiently absorbed by living organisms (Shannon et al. 1970; Skwarzec et al. 2012). ^210^Po is estimated to account for about 7% of the total effective dose to humans from ingested natural internal radiation (UNSCEAR 1988). The main source of ^210^Po in the atmosphere is the decay of ^222^Rn within continental landmasses (Jaworowski 1982; Jaworowski and Kownacka 1976). These radionuclides precipitate with rain and snow, depositing on land and water surfaces, including crops. Atmospheric deposition ^210^Po varies widely, from 0.05 to 0.5 kBq·m^−2^·y^−1^ ((Moore et al. 1976; Rangarajan et al. 1976). Plants absorb polonium indirectly through their root systems and directly from atmospheric fallout (Popova and Taskaev 1980). In marijuana (Cannabis sativa), direct deposition of ^210^Po on leaf surfaces is the primary method of accumulation (Mussalo-Rauhamaa and Jaakkola 1985; Skwarzec et al. 2001). Furthermore, these plants can be contaminated by ^210^Po found in certain fertilizers, especially phosphate fertilizers (Alam et al. 1997; Boryło et al. 2013a, b; Hussein 1987; Roessler et al. 1979).

Cannabis sativa is a resinous plant, enhancing its ability to absorb both wet and dry deposition on its leaves and other parts. These properties are likely due to the leaf structure and the distinct trichome layer. This plant is often used in the phyto-extraction of heavy metals from the soil due to its characteristics such as short vegetation time and rapid growth rates. (Boryło et al. 2013a, b; Długosz-Lisiecka 2016; Dushenkov 2003; Tso et al. 1966).

In industrial cultivation, *Cannabis sativa* is grown in fields ranging from several to several dozen hectares, impacting the plant’s structure. Cannabis can grow up to 3 m tall (depending on species and weather conditions), with reduced lower layers of leaves. During their 4–5-month growth period, they require substantial amounts of water and soil rich in organic carbon, phosphorus, and nitrogen. Consequently, the fields are heavily fertilized, particularly with phosphogypsum and nitrogen fertilizers. Numerous publications note that fertilizers, especially phosphate rock, significantly enrich the soil with polonium and radio lead precursors like radium or uranium. (Alam et al. 1997; Boryło et al. 2013a, b; Moore et al. 1976; Olszewski et al. 2015).

There are several types of cannabis products available on the market. One product, in particular, are prominently advertised: *Cannabis sativa* drought (hemp), Hemp is gaining more followers, with an increasing number of people using hemp-derived products. Cannabis available on the market is distinguished by name or CBD content due to various factors. Each trade name relates to the producer and species of *Cannabis sativa* from which it is derived. The amount of CBD or THC is not a reliable reference as these factors vary yearly; each batch of cannabis is tested immediately after harvest. The higher the CBD content, the more valuable the hemp plant and its market value. The THC content is controlled to ensure it does not exceed 0.2%. When a batch does not exceed 0.2% THC, it is released to the market; otherwise, it is destroyed. *Cannabis sativa* is often confused with *Cannabis indica*. The former has different properties than the latter. They are distinct species with unique characteristics both as plants and as products.

Smoking cannabis can be done in several increasingly popular ways. One method involves using a small glass pipe. Another method is using a “joint” or “spliff,” where cannabis is rolled in a paper with a cellulose filter. A third method is the “bong,” a glass pipe with a water filter. Additionally, gaining popularity is vaporization, where a special device is used to heat the cannabis without burning it, with controlled temperature.

In this publication, all of the aforementioned methods of consuming cannabis have been examined in terms of the amount of polonium-210 ingested with the smoke.

## Materials and methods

The concentrations of ^210^Po were determined in 10 samples of cannabis hemp (referring to the dried flowers and top leaves of *C. sativa* L.). All these products are legally available in Poland. According to the packaging, the plants were grown in the European Union, although the specific country of origin is rarely indicated. During the analysis, pure dried hemp was used as a sample without mixing it with tobacco.

Samples weighing approximately 1–1.4 g were transferred to beakers, spiked with ^209^Po as a yield tracer (176.9 ± 5.2 mBq/ml, NPL Product Code – R33-02b-2014–80361-1), and digested in 65% HNO₃. After evaporation, the dry residue was dissolved in 20 cm^3^ of 0.5 M HCl. Polonium was then electrodeposited from this solution in the presence of ascorbic acid (to reduce Fe^3+^) onto a silver disk. The electrodeposition process was conducted at 90 °C for 4 h, allowing more than 99% of the polonium to be deposited on the silver.

The activities of ^209^Po and ^210^Po were measured using an alpha spectrometer equipped with semiconductor silicon detectors (300 mm^2^ active surfaces, resolution 20 keV). The counting efficiency was about 30%, and the lower limit of detection (LLD) was 0.3 mBq for ^210^Po for a counting time of 3000 min. Samples were measured for 2–14 days, with polonium recovery rates ranging between 60 and 90%. Analytical quality control was regularly performed through participation in IAEA inter-comparison exercises using reference materials (IAEA-327, IAEA-414, IAEA-TEL-2011–03, MODAS-2015). The accuracy and precision of the radiochemical method were estimated to be less than 8%. (Antoine and Grant 2017; Boryło et al. 2013a, b; Poursafa et al. 2012; Skwarzec 2002, 2021; Skwarzec et al. 2003; Skwarzec and Jakusik 2003; Wieczorek et al. 2020, 2022).

The annual effective radiation dose (Ed) from the inhalation of ^210^Po can be calculated using the following formula:

$$\text{Ed}=365\text{ days}\cdot \text{MT}\cdot \text{Ci}\cdot \text{F}\cdot \text{D}$$

where:

days: number of days in a year.
MT: annual mass of cannabis consumed by the user, for regular smokers (0.68 g/day).
Ci: activity concentration of ^210^Po in the analyzed samples (Bq/kg).
F: effective dose coefficient for adult standard smoking: 6.0·10^−7^ Sv/Bq for ^210^Po (ICRP 2012).
D: desorption factor (%).

The inhalation factor F (fast) AMAD (ICRP 2012; IRCP Publ. 119 2012) was used for the calculations, as the particle size in cannabis smoke was assumed to be similar to that in tobacco smoke. The same assumptions about smoking and particle size used for tobacco were applied. The particle size for tobacco smoke ranges from 0.21 to 0.4 μm, while, for cannabis, it is 0.43 μm. The methods of smoking these products are also similar. Often, users mix hemp products with tobacco to increase their volume during smoking (Cohn et al. 1986 Fernández Tena and Casan Clarà, 2012; Hrycushko 2008; ICRP 2012; ICRP Publ. 119, 2012; Keith and Derrick 1960; Nathan and Scobell 2012; Wieczorek et al. 2022).

### Combustion of samples

The cannabis was homogenized and then combusted in an appropriate manner simulating real conditions using a vacuum pump under negative pressure. Smoke samples were not collected due to the very small scale of combustion (simulating real conditions). The difference between the amount of ^210^Po in unburned herb and its amount in ash was measured. Additionally, the activity remaining on the filters (water and cellulose) was measured. Measurement of the amount of smoke was impossible due to the low mass of the samples. The standard temperature of cannabis combustion during this process is about 600–700 °C (Fehr and Kalant 1972). In the case of vaporization, the temperature was set according to the manufacturer’s scale on the device.

## Methods of sample combustion

### Smoking a cigarette, commonly known as “smoking a joint or spliff”

This is one of the most popular methods of consuming cannabis, particularly dried flowers, and to a lesser extent, hashish or kief. An empty cigarette with a filter (usually cellulose) filled with finely ground cannabis, packed to allow the cigarette to burn freely, is used for smoking. This method can also involve using a piece of rolling paper, which is a small piece of paper made from cellulose, rice straw, or hemp fibers, along with a cigarette filter or a piece of cardboard serving as a filter. Often, to mitigate the effects of THC and/or CBD and to save on cannabis, it is mixed with tobacco. This method is popular not only because of the ease of preparation but also due to the general availability of materials needed to create a ready-to-smoke joint.

For burning, standard, commercially available cellulose filters with a length of 1 cm and a diameter of 0.3 cm, as well as cellulose rolling papers, which are thin materials that hold the contents of the joint or cigarette, with a length of 11 cm, were used. Before commencing the radiochemical analysis, both matrices were tested and it was found that the ^210^Po concentrations were below the detection limit (thus for calculations, it was assumed they did not contain ^210^Po). A new rolling paper and filter were used for each sample. The rolled cigarettes were 7 cm long and had a diameter ranging from 0.3 (at the filter) to 0.5 cm at the end.

### Smoking from a glass pipe

This method has become particularly popular in recent years due to the availability of glass pipes in almost any grocery or liquor store. Similar to using a “joint,” the cannabis material is usually finely ground, pure dried cannabis or a mixture of dried cannabis and tobacco. Apart from the glass pipe itself, this method does not require any other materials or equipment for smoking. An important factor when consuming cannabis this way is the lack of a filter, which would otherwise filter out larger particles during smoking. As a result, even smoldering fragments of the smoking material can reach the lungs. During combustion, it is also noticeable that tar and unburnt fragments accumulate on the pipe. Glass pipes can come in various shapes and sizes.

For burning the samples, the most popular available model of a glass pipe was used, with a length of 7.5 cm and a diameter of 0.3 cm, where the test material was placed in the first 0.5 cm of the pipe with an approximately 50% larger diameter. To avoid possible contamination, a new pipe was used for each analyzed sample, which was previously rinsed with 3 M HNO_3_ to remove any polonium that might have adsorbed on the glass.

### Smoking with a water pipe, commonly known as a “bong”

This method has recently gained popularity and is relatively new. It is similar to smoking with a glass pipe, but it includes a water filter reservoir. During smoking, the pipe is not placed directly into the smoker’s mouth but is instead submerged in water. The reservoir can range from a few milliliters to several liters, but the standard capacity is between 100 and 500 ml. The water serves to clean out unburnt fragments, homogenize, and cool the smoke. The combustible material is usually pure dried cannabis, hashish, or kief. This technique is very similar to using a “shisha” or water pipe but differs in that it does not use glowing charcoal as a heat source. The plant material is ignited continuously during inhalation.

For burning the plant material, a glass device with a height of 30 cm and an outlet diameter of 3 cm was used. The capacity of the water filter was approximately 200 ml, but for research purposes, 100 ml of distilled water (with a conductivity of 0.05 μS) was used. In this sample, the concentration of ^210^Po was below the detection limit, so it was not included in the calculations.

### Vaporization

This is the newest technique compared to the previously mentioned methods of consuming cannabis, requiring access to a heating device known as a vaporizer. In recent years, this method has been gaining increasing popularity due to the greater availability of vaporizers in stores. These devices come in two basic types: stationary and portable. The operating principle of both is similar, but they differ in size and minor construction details, depending on the manufacturer. The fundamental principle, in contrast to burning the material, is heating it, which produces vapor instead of smoke, hence the name of the device (from the English “vapor”). Additionally, most devices allow precise temperature control for heating the material. The vaporizer purchased for the study had a temperature adjustment range from 30 to 230 °C, with a built-in ceramic heating system and a filtering screen.

## Results and discussion

According to the described procedure, 10 different types of hemp samples were prepared for analysis.

**Fig. 1:**
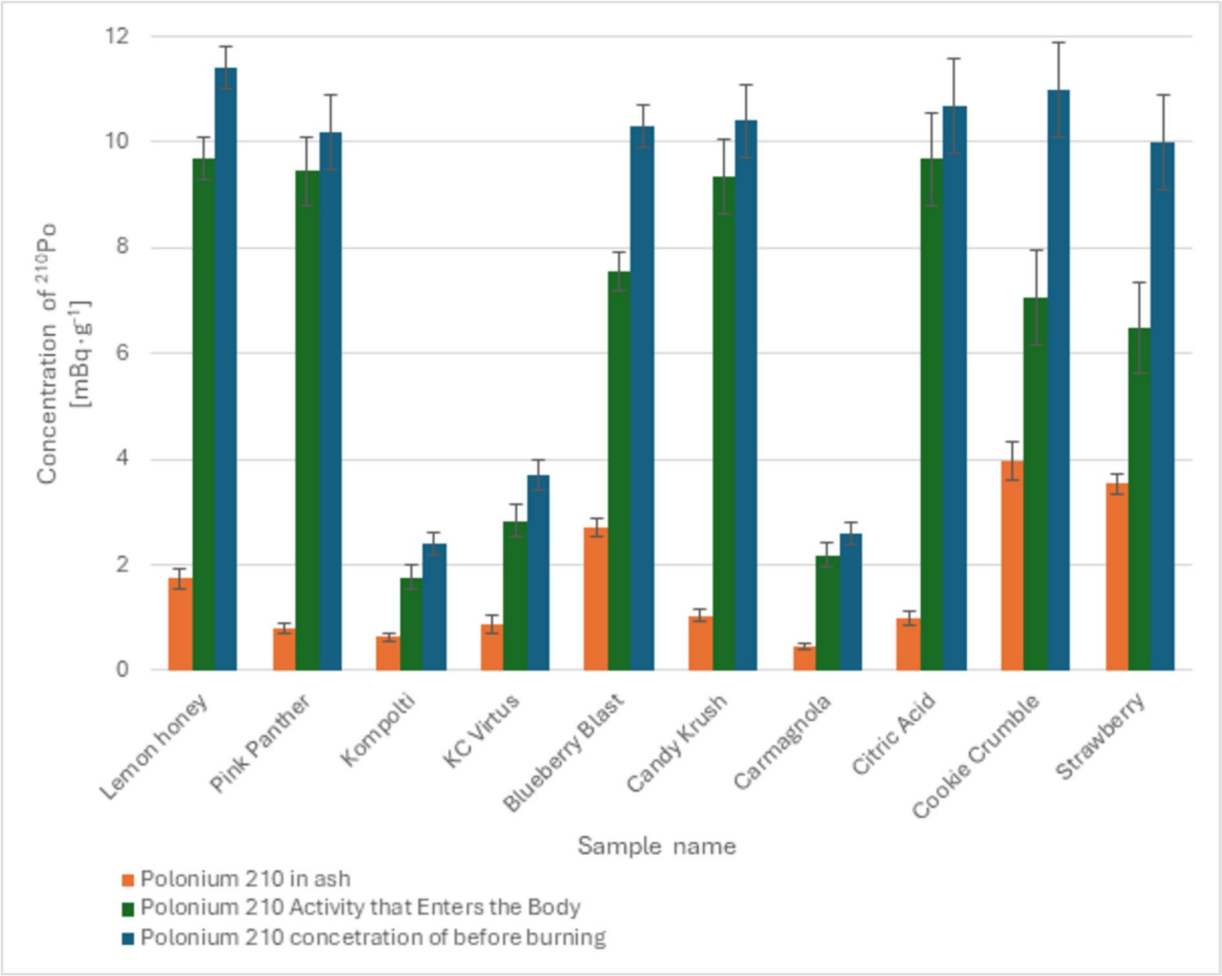
Desorbed activity, activity in ash after combustion and activity in the original sample during combustion from a glass pipe

The percentage of ^210^Po desorption that enters the bodies of individuals smoking dried hemp ranged between 64.0 ± 2.3% and 92.3 ± 1.7%. The design of the glass pipe does not include any filter; hence, the adsorbed ^210^Po entirely reaches the lungs of smokers along with the smoke. Based on the conducted study, it was estimated that an average of 79.4 ± 2.3% of ^210^Po is released during combustion. Detailed results of combustion using a glass pipe are presented in Fig. 1.

**Fig. 2:**
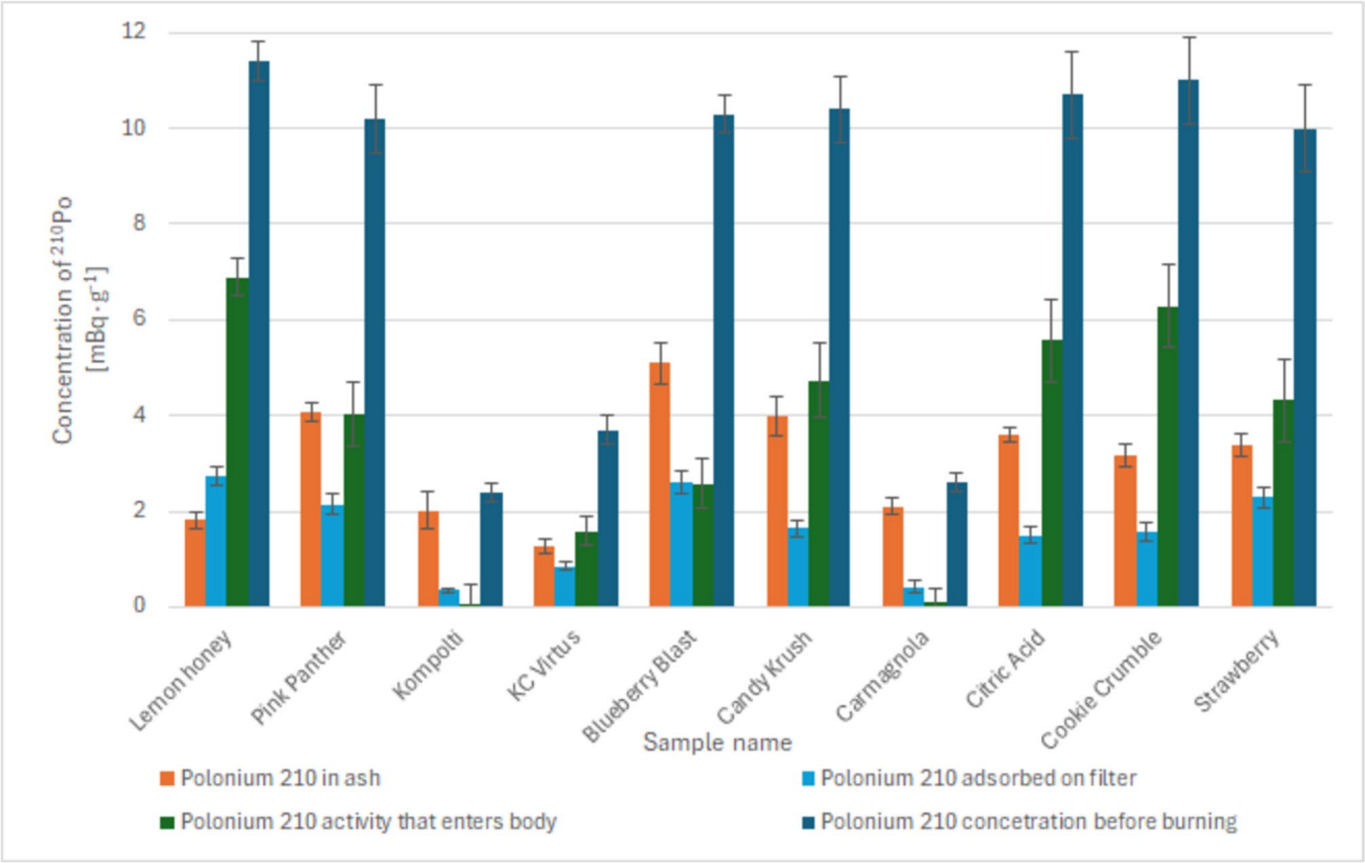
Desorbed activity, activity in ash after combustion and activity in the original sample during combustion from a cigarette

Polonium 210 was determined in the ash after combustion. The percentage of ^210^Po that enters the body with the smoke of smokers ranged from 1.7 ± 4.4 to 60.2 ± 1.1%. The efficiency of ^210^Po adsorption in the filter of a cigarette ranged from 14.1 ± 1.4 to 25.2 ± 1.5%. Detailed results of combustion using a cigarette are presented in Fig. 2.

**Fig. 3:**
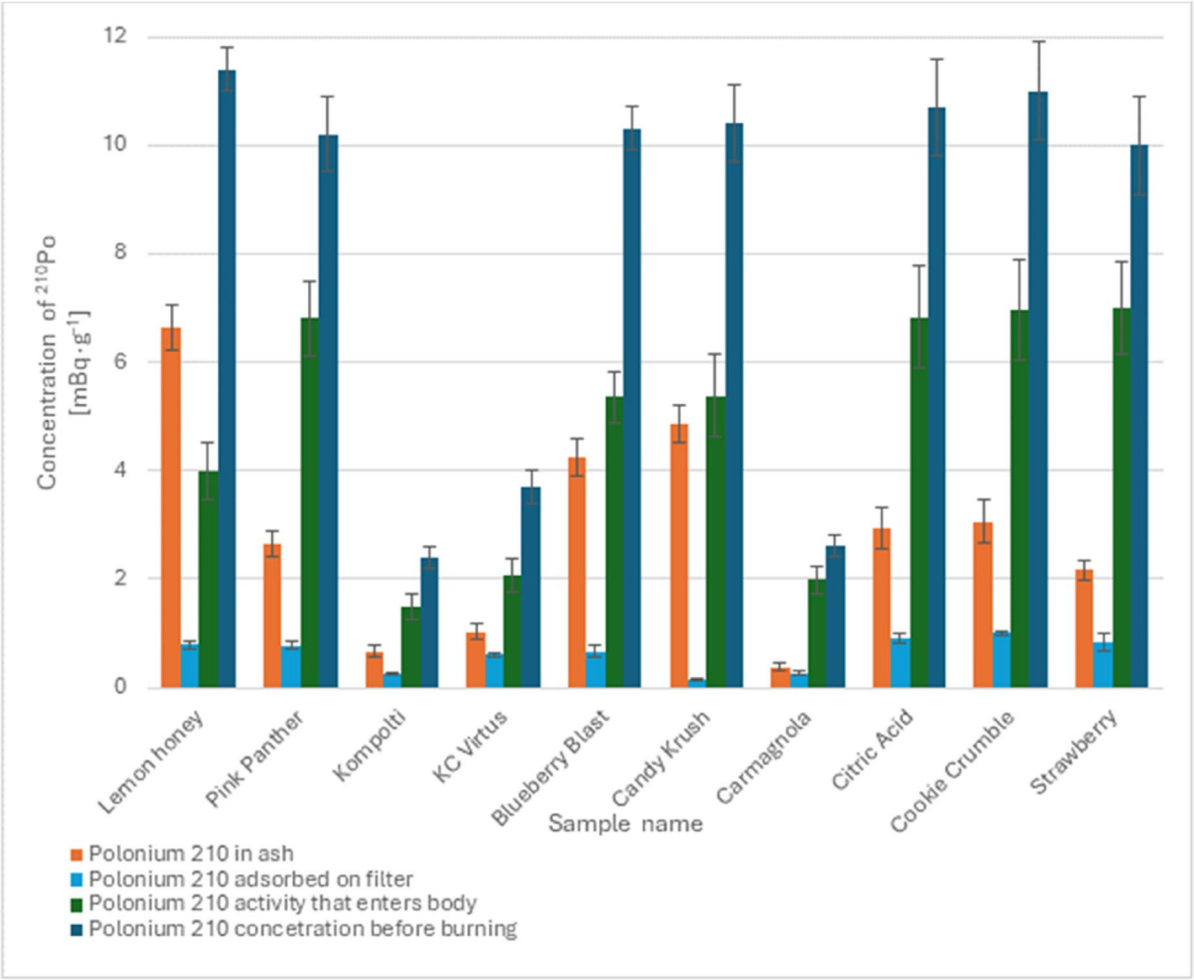
Desorbed activity, activity in ash after combustion and activity in the original sample during combustion from a water pipe

The ash from the combustion of hemp samples was subjected to radiochemical analysis. The percentage of adsorbed ^210^Po that enters the body with the smoke of smokers ranged from 30.1 ± 2.1 to 65.0 ± 2.1%. The adsorption of polonium ^210^Po in the water filter ranged from 1.4 ± 2.5 to 16.4 ± 2.2%. Detailed results of combustion using a water pipe are presented in Fig. 3.

**Fig. 4:**
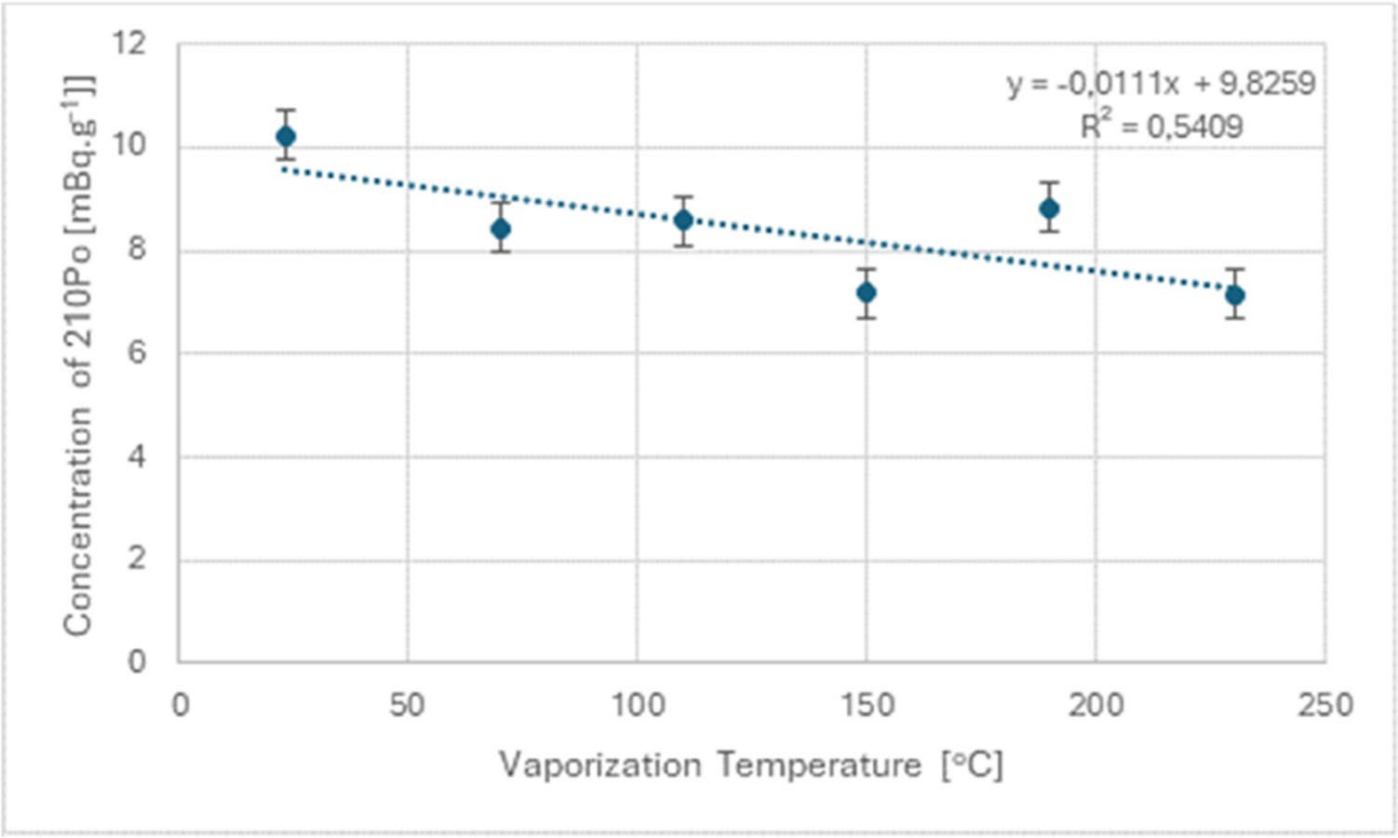
Graph showing the decrease ^210^Po concentration during vaporization

Vaporization was conducted at temperatures ranging from 23 to 230 °C (sample vaporized-Pink Panther). The rate of ^210^Po desorption with increasing temperature and the effectiveness of limiting desorption using this method were measured. The detailed decrease in concentration with increasing temperature is presented in Fig. 4.

### Radiation dose

Doses from smoking hemp are presented in Table 1. The smallest dose can be observed in the case of vaporization. This proves the advantages of this method over other smoking methods. Doses in this case were calculated with an average desorption between 150 and 230 °C. This is the recommended vaporization temperature. The highest dose was recorded in the case of smoking from a glass pipe without a filter. The dose was calculated according to the formula given in the materials and methods section.

**Table 1:** Radiation dose from hemp smoking

Sample	Glass pipe [nSv]	Cigarette [nSv]	Water pipe [nSv]	Vaporization with average desorption in the range of 150–230 °C [nSv]
Lemon honey	1.35	0.63	0.69	0.42
Pink Panther	1.21	0.57	0.62	0.38
Kompolti	0.28	0.13	0.14	0.09
KC Virtus	0.44	0.20	0.22	0.14
Blueberry Blast	1.21	0.57	0.62	0.38
Candy Krush	1.23	0.57	0.62	0.38
Carmagnola	0.31	0.15	0.16	0.10
Citric Acid	1.26	0.59	0.64	0.39
Cookie Crumble	1.30	0.61	0.66	0.40
Strawberry	1.18	0.55	0.60	0.37

When comparing the annual radiation doses from polonium-210 (^210^Po) due to inhalation from smoking cannabis and tobacco, the differences are striking. For cannabis, your data shows that the highest annual dose from inhaling ^210^Po is 1.35 nSv/y (for Lemon Honey using a glass pipe), with lower values observed for other consumption methods, such as 0.63 nSv/y for smoking a cigarette, 0.69 nSv/y for a water pipe, and 0.42 nSv/y for vaporization. These values, measured in nanoseiverts, indicate a very low level of radiation exposure from cannabis smoke.

In contrast, studies on tobacco show significantly higher doses. Ferri and Baratta report inhaled doses ranging from 1.700 to 4.000 nSv/y, which means the radiation exposure from tobacco is 1.700 to 28.000 times higher than for cannabis. Khater reports an even higher inhaled dose of 193.000 nSv/y, which is 143.000 times higher than the maximum dose from cannabis smoking. Similarly, Carvalho and Oliveira estimate annual lung doses from inhaled tobacco smoke at 970 to 1.520 nSv/y, again showing that tobacco results in radiation exposure that is 10,000 to 17,000 times higher than the doses associated with cannabis. Black and Bretthauer [V.10] also report inhaled doses from tobacco of 2.600 nSv/y, which is 1.900 to 28.000 times higher than the doses from cannabis smoke.

In summary, the radiation exposure from smoking tobacco is vastly higher than that from smoking cannabis, with differences reaching thousands of times in magnitude. Even the lowest reported doses from tobacco studies are significantly greater than the highest doses recorded for cannabis, demonstrating that cannabis poses a much lower risk of radiological exposure compared to tobacco, particularly when considering inhalation of smoke (Carvalho et al. 2017).

## Conclusions

The conducted study on the concentration of polonium-210 (^210^Po) in dried cannabis samples and various methods of its consumption provides significant insights into the potential health risks associated with radiation exposure. Here are the key findings:

Firstly, the analysis of 10 different dried cannabis samples revealed considerable variation in ^210^Po concentrations. The highest concentrations were found in the samples that had not undergone thermal processing, indicating the presence of polonium in raw plant materials. This underscores the importance of understanding the baseline contamination levels in cannabis before consumption.

Secondly, the study demonstrated that the method of consumption significantly affects the level of ^210^Po exposure. Smoking using a glass pipe resulted in the highest desorption of polonium (approximately 80%), while methods utilizing filters, such as smoking with a cellulose filter cigarette, showed lower desorption values. This finding is crucial as it highlights the differences in radiation dose received by users depending on their chosen method of cannabis consumption.

The effectiveness of various filters was also assessed, revealing that water and cellulose filters significantly reduced the amount of ^210^Po inhaled by users. The water filter was the most effective, reducing polonium desorption to about 8%, while the cellulose filter reduced it to approximately 20%. This suggests that implementing appropriate filtration methods can mitigate the health risks associated with ^210^Po in cannabis smoke.

Moreover, vaporization, as an alternative method of consumption, showed a temperature-dependent desorption rate of ^210^Po. Higher temperatures led to greater release of polonium into the lungs of users. This relationship was described by the linear equation: y =  − 0.0111 ×  + 9.8259 (*R*^2^ = 0.5409), indicating that temperature control is vital in reducing radiation exposure during vaporization. In conclusion, while vaporization presents a potentially less harmful method of cannabis consumption compared to smoking, it is not without risks. The desorption of ^210^Po at high temperatures underscores the need for informed consumption practices and regulatory oversight. As the use of vaporizers continues to rise, both consumers and manufacturers must be aware of the implications for radiation exposure and take steps to mitigate these risks. This study contributes to the ongoing dialogue about the safety of cannabis consumption methods and highlights the importance of ongoing research and regulation in this evolving field.

In light of the findings presented in this study, there is a clear need for regulatory measures addressing the concentration of polonium-210 (^210^Po) in cannabis products and the methods of their consumption. The study reveals significant levels of ^210^Po in cannabis, posing potential health risks, particularly when consumed through methods such as smoking with a glass pipe, which results in the highest desorption of polonium. The variability in ^210^Po exposure depending on the consumption method underscores the necessity for public awareness and education on safer consumption practices. Regulatory authorities should consider establishing maximum allowable limits for ^210^Po in cannabis products and promote safer consumption methods, thereby protecting consumers from unnecessary radiation exposure and ensuring public health safety.

## Supplementary Information

Below is the link to the electronic supplementary material.

Supplementary file1 (DOCX 24 KB)

## Data Availability

Data used in desorption calculations are available at link 10.6084/m9.figshare.26139766. The authors can also make the alpha spectrometry spectra and once other data, e.g., background measurements available on request but due to the nature of the data these can only be sent to the email address provided. The authors are willing to make any data available**.**
