# Supplementary material for: Quantifying of highly radioactive and radiotoxic polonium-210 intake from cannabis (Cannabis sativa L.): impacts of different smoking and vaporization techniques
Source: Environ Sci Pollut Res Int. 2024 Oct 15;31(51):61138–46. doi: 10.1007/s11356-024-35263-w (PMC11534889; doi:10.1007/s11356-024-35263-w)
Supplement: Supplementary file 1 — Supplementary file1 (DOCX 24 KB) [file 11356_2024_35263_MOESM1_ESM.docx]

Table 1 Samples of Dried Hemp Without Thermal Processing

| Sample | Mass of Burned Sample [g] | Concentration of ^210^Po [mBq∙g^-1^] |
| --- | --- | --- |
|  |  |  |
| Lemon honey | 1,48 | 11,4±0,4 |
| Pink Panther | 1,05 | 10,2±0,7 |
| Kompolti | 1,10 | 2,4±0,2 |
| KC Virtus | 1,08 | 3,7±0,3 |
| Blueberry Blast | 1,01 | 10,3±0,4 |
| Candy Krush | 1,02 | 10,4±0,7 |
| Carmagnola | 1,10 | 2,6±0,2 |
| Citric Acid | 1,02 | 10,7±0,9 |
| Cookie Crumble | 1,21 | 11,0±0,9 |
| Strawberry | 1,05 | 10,0±0,9 |

Table 2 Glass Pipe

| Sample | Mass of Burned Sample [g] | ^210^ Po Concentration in Ash [mBq∙g^-1^] | ^210^Po Activity that Enters the Body [mBq∙g^-1^] | Percentage of Activity that Enters the Body [%] |
| --- | --- | --- | --- | --- |
| Lemon honey | 1,10 | 1,74±0,18 | 9,70±0,39 | 84,79±1,17 |
| Pink Panther | 1,04 | 0,79±0,09 | 9,45±0,65 | 92,29±1,68 |
| Kompolti | 1,07 | 0,63±0,09 | 1,77±0,24 | 73,75±3,74 |
| KC Virtus | 1,04 | 0,87±0,16 | 2,84±0,31 | 76,55±4,38 |
| Blueberry Blast | 1,17 | 2,71±0,16 | 7,56±0,37 | 73,61±0,56 |
| Candy Krush | 1,08 | 1,04±0,11 | 9,34±0,70 | 89,98±1,60 |
| Carmagnola | 1,07 | 0,44±0,06 | 2,19±0,24 | 83,27±2,95 |
| Citric Acid | 1,12 | 0,98±0,14 | 9,68±0,87 | 90,81±2,71 |
| Cookie Crumble | 1,08 | 3,96±0,36 | 7,05±0,90 | 64,03±2,34 |
| Strawberry | 1,07 | 3,53±0,20 | 6,47±0,86 | 64,70±1,97 |

Table3 Cigarette

| Sample | Mass of Burned Sample  [g] | Concentration of ^210^Po [mBq∙g^-1^] | Activity of ^210^Po on the Filter After Combustion  [mBq∙g^-1^] | Activity of ^210^Po That Enters the Body [mBq∙g^-1^] | Percentage of Activity That Enters the Body  [%] | Percentage of ^210^Po Adsorbed on the Filter [%] |
| --- | --- | --- | --- | --- | --- | --- |
| Lemon honey | 1,69 | 1,82±0,17 | 2,73±0,19 | 6,89±0,39 | 60,23±1,13 | 23,86±1,29 |
| Pink Panther | 1,07 | 4,07±0,21 | 2,14±0,21 | 4,03±0,68 | 39,36±2,93 | 20,90±1,17 |
| Kompolti | 1,17 | 2,01±0,39 | 0,35±0,05 | 0,04±0,44 | 1,67±4,38 | 14,58±5,52 |
| KC Virtus | 1,03 | 1,26±0,14 | 0,86±0,10 | 1,59±0,31 | 42,86±4,69 | 23,18±2,46 |
| Blueberry Blast | 1,22 | 5,10±0,43 | 2,59±0,24 | 2,58±0,53 | 25,12±4,73 | 25,22±1,49 |
| Candy Krush | 1,15 | 3,99±0,40 | 1,65±0,18 | 4,74±0,79 | 45,66±3,60 | 15,90±2,09 |
| Carmagnola | 1,21 | 2,11±0,19 | 0,42±0,13 | 0,10±0,30 | 3,80±11,45 | 15,97±9,87 |
| Citric Acid | 1,13 | 3,59±0,14 | 1,50±0,17 | 5,57±0,87 | 52,25±2,49 | 14,07±1,36 |
| Cookie Crumble | 1,04 | 3,16±0,24 | 1,57±0,19 | 6,28±0,87 | 57,04±2,36 | 14,26±1,94 |
| Strawberry | 1,03 | 3,38±0,23 | 2,30±0,22 | 4,32±0,86 | 43,20±4,24 | 23,00±1,31 |

Table 4 Water pipe

| Name | Mass of Burned Sample [g] | Concentration of ^210^Po [mBq∙g^-1^] | Activity of ^210^Po on the Filter After Combustion [mBq∙g^-1^] | Activity of ^210^Po That Enters the Body [mBq∙g^-1^] | Percentage of Adsorbed ^210^Po That Enters the Body [%] | Filter Efficiency [%] |
| --- | --- | --- | --- | --- | --- | --- |
|  |  |  |  |  |  |  |
| Lemon honey | 1,01 | 6,65±0,42 | 0,79±0,07 | 4,00±0,53 | 65,03±2,06 | 6,91±1,12 |
| Pink Panther | 1,11 | 2,65±0,24 | 0,78±0,07 | 6,81±0,69 | 33,50±1,74 | 7,62±1,54 |
| Kompolti | 1,06 | 0,67±0,11 | 0,24±0,02 | 1,49±0,25 | 37,92±5,25 | 10,00±3,22 |
| KC Virtus | 1,07 | 1,03±0,14 | 0,61±0,04 | 2,07±0,31 | 44,20±3,83 | 16,44±2,16 |
| Blueberry Blast | 1,09 | 4,25±0,35 | 0,67±0,10 | 5,35±0,48 | 47,91±1,40 | 6,52±2,76 |
| Candy Krush | 1,23 | 4,86±0,36 | 0,14±0,02 | 5,38±0,77 | 48,17±2,48 | 1,35±2,46 |
| Carmagnola | 1,07 | 0,38±0,07 | 0,27±0,03 | 1,98±0,25 | 24,71±4,70 | 10,27±4,4 |
| Citric Acid | 1,03 | 2,93±0,38 | 0,90±0,10 | 6,83±0,94 | 35,93±3,39 | 8,44±2,77 |
| Cookie Crumble | 1,02 | 3,06±0,40 | 1,00±0,05 | 6,95±0,92 | 36,88±3,28 | 9,08±1,86 |
| Strawberry | 1,02 | 2,17±0,18 | 0,83±0,17 | 7,00±0,85 | 30,00±2,07 | 8,30±4,64 |

Table 5 Vaporization

| Vaporization Temperature [^o^C] | Concentration of ^210^Po [mBq^.^g^-1^] | ± |
| --- | --- | --- |
| 23 | 10,2 | 0,7 |
| 70 | 8,4 | 0,8 |
| 110 | 8,6 | 0,7 |
| 150 | 7,2 | 0,4 |
| 190 | 8,8 | 0,6 |
| 230 | 7,2 | 0,5 |
